# Women's perspectives on resilience and research on resilience in motherhood: A qualitative study

**DOI:** 10.1111/hex.13762

**Published:** 2023-04-10

**Authors:** Susan Hannon, Agnes Higgins, Déirdre Daly

**Affiliations:** ^1^ School of Nursing and Midwifery, Trinity College Dublin The University of Dublin Dublin Ireland; ^2^ Trinity Centre for Maternity Care Research (TCMCR), School of Nursing and Midwifery Trinity College Dublin Dublin Ireland

**Keywords:** mental health, motherhood, perinatal, postpartum, resilience, women's perspectives

## Abstract

**Purpose:**

Definitional perspectives and operational approaches to the concept of resilience vary within the literature; however, little is known of women's opinions on current resilience research, or the philosophical and methodological directions women believe such research should take. This research explored women's perspectives on resilience research in the perinatal period and early motherhood and sought their opinions on the ways in which they believe research should be advanced.

**Methods:**

Following ethical approval, online interviews were conducted with 14 ethnically and socioeconomically diverse women who were mothers. Findings from a concept analysis on resilience in pregnancy and early motherhood, conducted by the authors, were shared with women before and during the interview. Interviews were organised in sections corresponding to the findings within the concept analysis' four philosophical (Epistemology, Linguistic, Logic, Pragmatic) principles and thematically analysed.

**Results:**

Epistemology—Women endorsed a dynamic process definition, and viewed resilience as influenced by multilevel, multisystemic processes. Linguistic—Women viewed words such as ‘adaptation’ and ‘adjustment’ as being more active and empowering than the term ‘coping’ in relation to motherhood. Logic—Women were resistant to the predominant operational conceptualisation of resilience as illness absence. Pragmatic—Women were wary of resilience research being used to reduce mental health support for other mothers and families.

**Conclusions:**

Women provided constructive criticisms on the current state of resilience literature. Women suggested actionable ways in which research may be developed to better align with the epistemological and ethical approaches women want to see in resilience and maternal mental health research.

**Patient or Public Contribution:**

Women who are mothers and participants in health research were consulted on their views of trends in mental health and resilience research in motherhood.

## 1 INTRODUCTION

There is an ongoing philosophical shift in approaches to mental health research as researchers challenge the prevailing focus on psychopathology in which health is, by default, defined as the absence of an illness or disorder.^1^ Included within this shift from pathology to a strengths‐based investigation, is the concept of resilience.^2^ Although definitions and operationalisation of resilience vary, there is some consensus in the broad understanding of resilience as ‘positive adaptation despite adversity’.^3^ ^,p.739^

Within the maternal mental health literature, the concept of resilience has enjoyed rapid growth in research interest over the past 2 years.^4^ Resilience is a logical avenue of mental health investigation considering that women are at greater risk of developing a mental health problem (MHP) in the first postpartum year than they are pre or during pregnancy,^5^ and the negative impact of MHPs on child development.^6^, ^7^ Additionally, resilience is a point of interest as several studies demonstrate higher levels of anxiety and depression years into motherhood compared to the first postpartum year.^8^, ^9^, ^10^

**Figure 1:**
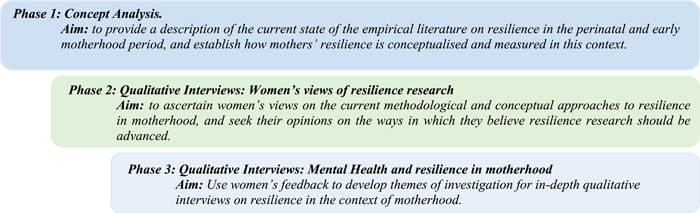
Research phases.

Despite increased interest in resilience in motherhood, women's perspectives on the varying definitions and approaches to measurements are distinctly absent from research.^4^ Few studies centre on women's voices and active participation in mental health or resilience research in motherhood,^11^ and, in general, little is known of the mental health research that women wish to see conducted.^12^ This is concerning as research shows substantial disparities between the health services offered and the supports women wish existed to meet their needs.^13^, ^14^ This gap illustrates the need for women to hold an active role in maternal mental health and resilience research, as failure to integrate the community's/research participants' perspectives is likely to result in findings that reflect the priorities, biases and worldviews of the researcher(s) rather than the needs of those whom the research concerns.^15^ The current paper focuses on the second of a three‐phased research design (Figure 1). Approaches to resilience differ according to context; however, the extent to which resilience research in motherhood follows or deviates from trends in resilience research in other contexts was unknown. Therefore, the first phase comprised a context‐specific concept analysis that described the current state of the literature on resilience in the perinatal period and motherhood (defined as up to 5‐years' postpartum).^4^ The concept analysis used a principle‐based framework^16^ which evaluated data according to four philosophical principles: Epistemology, Linguistics, Logic and Pragmatism. The analysis identified that in the context of the perinatal period and early motherhood, (i) research frequently operationalised resilience through illness absence, (ii) there was interchangeable use of associated concepts such as ‘coping’ and ‘adaptation’, (iii) measures of positive adaptation were predominately related to the mothering role and (iv) there were few qualitative explorations of women's resilience.^4^

The concept analysis was conducted with the intention of informing the current phase of the research. This phase aimed to (i) ascertain women's views on the current methodological and conceptual approaches to resilience in the perinatal period and early motherhood, and (ii) seek their opinions on the ways they believe resilience research should be advanced. Data from the current phase of research is intended to inform (i) researchers of the objectives that women wish to see included in resilience research, and (ii) the development of themes of investigation for in‐depth qualitative interviews on resilience in the context of motherhood.

## 2 METHODS

### 2.1 Participants

The university's Research Ethics Committee granted ethical approval. Participants in the longitudinal Maternal health And Maternal Morbidity (MAMMI) study were invited to join online one‐to‐one interviews. Participation was open to all consenting participants in the study without exclusion criteria. Recruitment took place over two periods; November 2020 and June 2021. Women received a participant information leaflet, a hard‐copy consent form for their own records and a link to an online consent form, a summary of the concept analysis findings from phase one and a semistructured interview guide. Seven women were interviewed following the first recruitment invitation which used convenience sampling. However, it became apparent that a majority (not all) of respondents to the first invitation were White‐Irish, partnered or had a third‐level education. Therefore the researchers issued a second invitation using purposive sampling to recruit women from more ethnically and socioeconomically diverse groups. Participants self‐identified if they belonged to one or more of the following groups: ethnic minority (Irish traveller, Black, Asian, ethnic minority groups), migrant (of any ethnicity), seeking/received asylum or refugee status in Ireland, 30 years of age or younger when they had their first child, do not have a graduate (third level) education, experiencing or experienced homelessness in the past 7 years, LGB identifying and single mothers. Seven women were interviewed during the second recruitment period. The participants' children were aged 6 months to 7 years old. Fourteen participants consented to the interview (Table 1).

**Table 1:** Participants' characteristics.

Current partner status	*n*	Ethnicity	*n*	Number of children	*n*	Sexual orientation	*n*
Heterosexual relationship	11	White Irish	8	One child	5	Bisexual	2
Same‐sex relationship	1	White European	4	Two children	2	Not disclosed	12
Not in a relationship	2	South‐East Asian	1	Three children	3		
		Mixed ethnic background	1				

### 2.2 Procedure

The research used a qualitative descriptive design. Data were collected using one‐to‐one semistructured interviews conducted via Microsoft Office Teams or telephone depending on the preference of each participant. The interview guide was developed by the researchers and corresponded to the findings from each of the four (Epistemology, Linguistic, Logic and Pragmatic) principles of the concept analysis (Table 2). The key findings under each principle were shared with participants, and then they were asked related questions from the interview guide. Participants confirmed that they had shared all they wished to say before the interviewer moved to each consecutive principle. Participants were encouraged to ask questions at any point in the interview. One author (S. H.) undertook all interviews, which averaged 1 h and 18 min (range: 44 min to 1 h 49 min).

**Table 2:** Lay synopsis of findings from resilience in pregnancy and early motherhood concept analysis and interview guide.

Philosophical principle	Lay synopsis of concept analysis findings derived from Hannon et al.^4^	Interview guide
*Epistemological findings*: *How is resilience defined?*	Most often resilience was defined as a trait: trait definitions approach resilience as a set of personal/internal traits which are a stable feature of someone's personality, and these traits help someone to be resilient when they are faced with challenges. In this approach, resilience is often measured using a scale. There is a lot of good research to show how certain aspects of personality are associated with better mental health outcomes during or after adversity. But, it may be difficult to develop resilience‐based interventions that can be used with a large number of people if resilience is considered related to individual personalities. Resilience was sometimes defined as a process: process definitions consider resilience to be an ongoing process influenced by multiple individual, contextual, familial, social, environmental, political, economic and cultural factors. These approaches sometimes look at mental health outcomes (low psychopathology, high mental well‐being) and/or positive adaptation outcomes (functionality, competence, etc.). The rest of the studies provided an explanation of how they would measure resilience (usually as stable levels of depression or anxiety over time), or did not give a definition of resilience.	*What are your thoughts on these definitions and perspectives?* *Do you agree/disagree with these definitions?* *How do you define resilience?* *What perspective should researchers take in resilience research?*
*Linguistic findings*: *What kind of language is used in resilience research?*	In the perinatal and early motherhood literature, the terms coping or coping strategies, adaptation and adjustment, protection and resistance were commonly used or associated with resilience.	*What do you think about these terms?* *How do you feel they fit into the concept of resilience?*
*Logical findings*: *How is resilience in motherhood measured?*	(i) Resilience scales: Resilience scales are usually used where researchers take a trait approach to resilience; however, they are rarely used alone and are often used alongside mental health outcome measures. (ii) Mental health outcomes: Depression was the leading mental health outcome of interest in the maternal literature, followed by stress disorders such as PTSD, and anxiety. In most cases, low symptomology or illness absence is considered indicative of resilience as this is an ideal outcome, especially in contexts of adversity. Some studies also included measures for mental well‐being, quality of life, self‐compassion or psychological flexibility. (iii) Positive adaptation outcomes: Positive outcomes in the perinatal period and early motherhood literature frequently related to a woman's adaptation and competence in the parental role, such as parenting sense of competence or family functioning.	*What are your thoughts on the ways that resilience is currently measured?* *How would you like to see resilience measured in future research?*
*Pragmatic findings*: *How are the findings from resilience research in motherhood being applied to practice?*	*Usefulness to research*: None of the included studies presented women's perspectives on how resilience should be defined or measured in the perinatal period and early motherhood. *Usefulness to clinical practice*: The concept analysis did not find any examples of resilience research used in clinical practice. Though several authors made suggestions as to how their findings might be implemented.	*What are your thoughts on the ways that resilience has been used in research?* *What are your thoughts or suggestions for resilience in practice?* *How would you like to see the concept of resilience used in research and practice?*

### 2.3 Data analysis and rigour

Interviews were audio‐recorded, transcribed, pseudonymised and thematically analysed using Braun and Clarke's methodology^17^ and managed using Microsoft Excel. Analysis was conducted concurrently through data collection to encourage an iterative interaction between the data and analysis.^18^ To ensure coding consistency and agreement, all researchers independently coded two interviews from each recruitment period, compared and refined codes and themes and discussed confirmatory and negative cases.^18^ The data were analysed in sections that corresponded to participants' responses to the findings of each principle (Epistemology, Linguistic, Logic and Pragmatic) of the concept analysis. Findings are presented accordingly.

Each participant received a synopsis of the interview findings, supported by anonymised quotes. They were asked if they felt that the findings were representative of their views and/or if they felt that the researchers had under‐ or overemphasised findings. This step further integrated participants' involvement within the research process to ensure the credibility of the findings through member checking.^19^ Participants received the synopsis via email and a posted hardcopy, and were invited to respond in any way that was most convenient to them (e.g., email or post replies). Four women responded to add to or clarify their views on the research. For example, one woman suggested that the tensions that arise from navigating cultural differences as a migrant woman and mother living in Ireland were an issue of personal importance and suggested it as an avenue for further research. Each participant confirmed that the findings were representative of their views and some mentioned that they felt that the collated findings had captured some issues which they themselves had not given voice to in the interview. Participants' identities have been anonymised for publication and illustrative quotes are presented using pseudonyms.

The researchers are maternal health researchers with backgrounds in psychology, midwifery and mental health nursing. All acknowledge that they are advocates of women's health, participatory/advocacy approaches in research design and conduct and the centrality of women and their voices.

## 3 FINDINGS

### 3.1 Women's perspectives on the Epistemological findings

Most women conceptualised resilience as a complex, multidimensional construct involving the individual and their lived experiences, influences from family, religion and culture and social and professional support. Resilience factors described at the individual level were: optimism, positivity, confidence, emotional intelligence, problem‐solving abilities, help‐seeking behaviours, a sense of purpose, a drive for independence and a strong work ethic. Although some women initially expressed trait views on resilience, their perspectives shifted as they developed their thoughts, for example, one woman questioned her original conception of resilience as ‘just getting on with it’ (Keva), as she reflected on her experiences:

Was it because I thought I was being resilient, that it led to that? That maybe if I had asked for more help from the beginning, it wouldn't have got to that? Say the downfall in my mental health because I was so used to being the person who was like ‘Oh, it's grand! We can do it, it's fine, we don't need any help. I'm really strong, I'm well able for this’. And then… I wasn't. (Keva)

Overall, a majority of women felt that a dynamic perspective most accurately captured the meaning of resilience. The largest portion of the women's conversation related to factors external to the individual. Several described how upbringing, family and culture provided a source and an exemplar of resilience.

I don't think we have inherent traits like that. I think it's all about our experiences and the way that we have been parented. And then when you are actually parenting, the kind of supports that you have. So I don't… (believe that) resilience is something that some women just have and some women don't because (of) their personality. (Saoirse)

For me, resilience really has been impacted and influenced heavily from a family, cultural, ethnic and religious point of view. And that's from my upbringing and the community I've grow up and into, and the parental teachings. (Aashvi)

Women were surprised that the studies included within the concept analysis favoured a trait definition, as they conceived motherhood as an ongoing transition involving multiple periods of adaptation and adjustment. Women stressed the importance of understanding resilience in the context of the availability of multiple layers of social support and the types of support each layer brings. For example, sources of support were partners, siblings, parents and in‐laws, friends, peer groups, healthcare professionals (HCPs) and social protection systems (housing authorities and charities, domestic abuse support and legal aid). The types of support offered were emotional, practical, informational, resource access and financial. For some, timely access to essential support determined the outcome of their experience.

If they hadn't opened their doors to me, I think, yeah, it would have ended very differently. And so yes, it all came together in the perfect, kind of positive storm. (Sana)

In fact, several women viewed resources and support available as key determinants of their resilience:

(I) tried to think about whether or not I was resilient; I have had a very satisfying mothering journey. But I know that that's because of the supports that I have. I don't feel like I'm more resilient than anybody else, I think I've been able to be resilient because of everything that I have. (Saoirse)

### 3.2 Women's perspectives on the Linguistic findings

**Figure 2:**
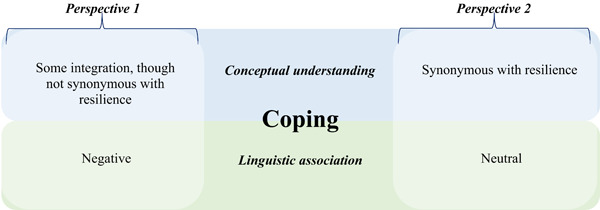
Two perspectives on the linguistic association and conceptual understanding of ‘Coping’.

The linguistic findings generated discussion around the linguistic and conceptual uses of the word coping. Two perspectives became apparent: the first perspective regarded coping as having negative linguistic connotations, though conceptually integrated within the larger concept of resilience. The second demonstrated neutral associations and was viewed as synonymous with resilience (Figure 2).

#### 3.2.1 Perspective 1: Coping: Linguistically negative, conceptually integrated

Seven women viewed the word coping as having negative connotations, particularly in connection with motherhood. Some commented that coping was often framed as a question linked to a woman's ability as a mother, and carried a sense of judgement.

It's clearly used like… a real hush, hush, ‘Oh, she's not coping’, you know? And it's clearly a negative, ‘Oh, she's not doing well with this; she's not able to do it.’ And it's kind of demeaning if you look at it really, because no one would… you wouldn't ring your Mam and say ‘Oh god yeah, I'm coping really well with this.' It's not something I think people really use to describe themselves, but I think it might be used as something people use to describe others, as a negative thing. (Keva)

Women considered coping, poised as a question, as restrictive. They felt there was only one acceptable response, which potentially hindered women's openness about the difficulties of parenthood.

It's not ok as a mother to say that you're not coping, where (it might be seen) that you're not doing a good enough job. (Saoirse)

From this viewpoint, coping was seen as a short‐term strategy offering temporary distress alleviation; coping was viewed as adequate for survival but inadequate for living or thriving, whereas resilience was aligned with long‐term solution finding.

I don't think that coping is the same as resilience, coping is… barely scraping by, coping is surviving…. Coping has to do with staying barely above the water. Resilience is being able to swim. If you cope, you can fight off the waves and you can breathe, but you're not alive in any sort of way, and you're not about to become better or you're not about to fix the situation or handle the situation. (Sana)

Women were of the view that a focus on coping creates a perception that researchers and HCPs expect women to find or learn ways to cope by themselves without the involvement of external support.

If you are asking people to constantly just cope, you are not getting to the root of the problem. If you're trying to get people to just focus on coping strategies, you're kind of missing the point of resilience a bit. (Sadhbh)

#### 3.2.2 Perspective 2: Coping: Linguistically neutral, conceptually synonymous

Within the second perspective, women did not speak about coping as having positive or negative linguistic associations; rather they spoke directly about the conceptual integration of coping with resilience, which was synonymous.

Resilience is, to me, means… coping without breaking. That's my thing, if I have an image, not necessarily in motherhood but generally, as in being strong… despite possible circumstances that are not necessarily positive or not necessarily nurturing. (Eleni)

Women preferred the use of the words adaptation and adjustment. They felt these words encompassed a more compassionate view of motherhood as a transition that takes time. All women were of the view that the language used around resilience and women's mental health in motherhood should never suggest deficit, failing or personal responsibility for mental health challenges.

Something like adaptation makes it… gives you a lot more power in the situation because then you're able to change and make changes that you need. (Saoirse)

It might just take some people a little longer to adjust to it, you wouldn't like them to be labelled as being… you don't want someone to be labelled as weak because (they need) a bit longer to being used to be a mum, do you know what I mean? That's the sort of linguistic concern. (Evelyn)

### 3.3 Women's perspectives on the Logical findings

Women were of the opinion that, although descriptions of mental ill‐health can assist in building an understanding of resilience, conceptualising resilience solely as the absence of mental distress does not reflect women's lived experience of resilience. Several women mentioned their own mental health and spoke of their resilience in spite of mental health challenges. The idea that mental ill‐health can co‐exist alongside resilience was a strong and reoccurring message from women:

I think to say that mental ill‐health has anything to do with the, the grade of resilience that you display….I don't think just because somebody comes out with a scratch means that they have a lower level of resilience; I don't think that would be right. At least definitely, for myself, I think my resilience levels are a lot higher than my mental health levels are. (Sana)

I don't think one thing invalidates the other, per say. So I think resilience and mental health can co‐exist the same way that a person with a chronic illness can still be healthy as long as the chronic illness is maintained. So, I don't think one thing invalidates the other. (Inés)

Overall, women preferred the method of combining mental health measures with positive indications of well‐being in quantitative research to encompass a fuller understanding of resilience and mental health in motherhood.

If you measure just my depression, you don't see the positive outcome, that I am able to, despite my mental health issues, or despite the, abuses I've suffered in my life. I'm still able to create positive outcomes, positive adaptations, positive… a positive life for my children; they don't know or ever feel that my mental health is less than, maybe perfect. … if it's looked at, it needs to be looked at from both sides. (Sana)

Some women commented that although women's adaptation to the mothering role might be an expected and logical area of investigation for resilience, they were wary that this approach would reduce women to a functional role while neglecting the wholeness of the women or other facets of their well‐being and fulfilment.

Women gravitated towards positive indications of well‐being when asked how they would like to see resilience investigated in future research. Themes of ‘Creativity’, ‘Nurturing a Sense of Self and Identity’ and ‘Career or Personal Goals’ were three interconnected areas that women suggested could be used to provide a fuller understanding of mental health and resilience in motherhood. Creativity included (i) maintaining, or taking up new hobbies or interests; which further supported social relationships and identity independent of the mother role, and (ii) engaging in activism; which supports social interaction and a sense of purpose. For some women, career and personal goals played a significant role in their identity, and stepping away from the career facet of their identity was perceived as a loss of self and creativity. The benefits of an expanded exploration of women's resilience were reiterated in women's responses to the study's findings, for example, that future research might reconsider: ‘How resilience is measured with an emphasis on a woman's life outside of motherhood’ (Sadhbh).

### 3.4 Women's perspectives on the Pragmatic findings

In relation to the application in research, all women endorse efforts to conduct research that centres women's voices as the experts of their lived experiences. They were of the view that inclusion indicated respect and a progressive approach to research.

The participants should be central, you know? You asking me what do you think resilience is, rather than getting a definition from a book, textbook or whatever and applying it to the woman…. So, I don't agree with the other kind of… more old fashioned kind of research. (Eleni)

Although women were interested in resilience research being translated into practical and educational supports for women as mothers, some were wary that research on resilience might be used to justify policy decisions in which resources and supports could be removed for mothers and families.

There's a bit of me that feels that… if you're trying to instil resilience in mothers, it's because you're not going to help them in other ways. Now, I know obviously that's not where your study is coming from, but my first reaction to that word ‘resilience’… (it) always feels like it's putting the responsibility on the mother. And it feels kind of like another, just another responsibility that's being put on us. (Saoirse)

The concept analysis did not reveal any study actively employing resilience or a resilience scale as a method of assessing or screening mental health in the postpartum period or early motherhood, however, it was presented as a suggestion by several authors. Many women questioned the feasibility of mental health assessments at all (regardless of whether the tools used in the assessment are for ill‐health or resilience) for two reasons: first, women frequently noted that the interactions they had with HCPs were not amenable to assessment or screening as appointments were short, and focused on their child's health. Second, a number of women described the severe lack of quality, timely service response following disclosure of mental distress.

## 4 DISCUSSION

Few studies centre women's voices and active participation in mental health or resilience research in motherhood,^11^ and, there are concerns that this absence may exacerbate gaps between the health services available and the health services that women actually need.^12^ Additionally, the absence of public and patient voices as important stakeholders in health research raises concerns about producing research that meets the public's needs.^15^

Indeed, the current research illustrates the disparity between the ways in which resilience in motherhood is presently researched and the ways in which women believe resilience should be researched.

Women engaged deeply in appraising empirical research and provided constructive criticisms on the current state of the literature and suggestions for pathways for developing future investigations. Although women identified a number of personal factors they perceived as associated with resilience, they were concerned that adoption of the trait perspective alone was reductive. This echoes the ethical concerns around trait conceptualisations in the literature.^20^ The findings show that women support a biopsychosocial‐ecological approach to resilience.^21^ In line with this approach, women considered the interactive roles of family, community, culture, and religious upbringing salient areas of investigation. Women highlighted the need for inclusive or holistic investigation and identified that factors and processes of resilience should not be viewed in isolation. Women were confident that future research should explore the many contextual, social and economic determinates of resilience. Socioeconomic factors associated with service provision and access were frequently commented on; being able to access the right resources (mental healthcare, housing, domestic abuse supports, community supports) at the right time was either instrumental or detrimental to a woman's mental well‐being. A recent scoping review of 17 studies applying resilience theory to the transition of parenthood found that though resilience support factors are explored more commonly at the personal level, headway is being made in mapping some interpersonal and contextual factors that support resilience in the parenthood transition.^22^

The findings illuminate the scope for development beyond resilience. Coping is a substantial concept of research within maternal mental health. There is recognition within the literature that some women with mental illness feel pressured to present an appearance of ‘coping’ due to fears that their child will be removed from their custody.^23^ To the authors' knowledge, there is no published literature on how women, with or without mental ill‐health, feel about HCPs' and researchers' use, and investigation of, coping. This provides a moment of insight for clinicians and researchers alike, as some women experience inquiries of ‘coping’ as judgement‐laden surveillance.

Women raised some concerns that resilience research could be used to burden women with responsibility for their experiences of distress and with responsibility for finding and accessing necessary support. At the same time, women were eager for this topic to be researched. Consistent throughout discussions was women's rejection of the consideration of maternal mental health in simplistic terms. Women's preferences for future research leaned towards qualitative and mixed methods research. There was a sense that surveys and questionnaires attempt to produce a ‘one‐size fits all’ understanding of resilience and mental health in motherhood, which risks missing the nuances of individual experiences and minimising women's needs. Women felt that a greater focus on women's experiences and their inclusion within research not only benefits data acquisition but benefits women.

### 4.1 Limitations

The study included participants for whom English was not their first language, however, the study's structure potentially excluded women who did not feel that they possessed sufficient English skills. Additionally, the research collected minimal socio‐demographic information from participants, and while the study included women of differing educational levels and backgrounds, most disclosed having a third‐level education qualification. Therefore, the findings should be contemplated in this light, and consider that women who have fewer socioeconomic resources to avail of further education may interpret resilience differently or hold different views as to how resilience should be researched.

## 5 CONCLUSION

This research reveals an opportunity for future investigations and investigators to shift the focus from individualised and pathologised explanations of resilience in a maternal context to developing a holistic, multilevel understanding. Women reject trait interpretations of women's resilience in motherhood as reductive and invalidating to the lived mental health and mental ill‐health experiences of motherhood. While they support the investigation of resilience as a dynamic process involving interacting influences from personal, familial, cultural and social domains, they also are cautious of an interpretation that may place a greater burden or responsibility on women. Women wish for future research to centre women's involvement in the research and respect the nuances of women's experiences of resilience and mental ill‐health, without judgement, during this life transition.

## CONFLICT OF INTEREST STATEMENT

The authors declare no conflict of interest.

## ETHICS STATEMENT

This study was approved by the Research Ethics Committees of Trinity College Dublin and the Ethics Committees of the three hospital recruitment sites: Rotunda Hospital Dublin, Coombe Women and Infants University Hospital, and Galway University Hospital. Informed consent was obtained from all individual participants included in the study.

## Data Availability

The anonymous transcripts that support the findings of this study are available from the corresponding author upon reasonable request that does not contravene the informed consent forms signed by the participants.
